# Management of war-related vascular injuries: experience from the second gulf war

**DOI:** 10.1186/1749-7922-8-22

**Published:** 2013-07-01

**Authors:** Ali Jawas, Alaa K Abbas, Munier Nazzal, Marzoog Albader, Fikri M Abu-Zidan

**Affiliations:** 1 Department of Surgery, College of Medicine and Health Sciences, United Arab Emirates University, PO Box 17666, Al-Ain, United Arab Emirates; 2 Department of Surgery, Mubarak Al-Kabeer Teaching Hospital, Jabriya, Kuwait; 3 Division of Vascular and Endovascular Surgery, University of Toledo Medical Center, Toledo, Ohio, USA

**Keywords:** Injury, Management, Trauma, Vascular, War

## Abstract

**Aim:**

To study the biomechanism, pattern of injury, management, and outcome of major vascular injuries treated at Mubarak Al-Kabeer Teaching Hospital, Kuwait during the Second Gulf War.

**Methods:**

This is a descriptive retrospective study. War-related injured patients who had major vascular injuries and were treated at Mubarak Al-Kabeer Teaching Hospital from August 1990 to September 1991 were studied. Studied variables included age, gender, anatomical site of vascular injury, mechanism of injury, associated injuries, type of vascular repair, and clinical outcome.

**Results:**

36 patients having a mean (SD) age of 29.8 (10.2) years were studied. 32 (89%) were males and 21 (58%) were civilians. Majority of injuries were caused by bullets (47.2%) and blast injuries (47.2%). Eight patients (22%) presented with shock.

There were 31 arterial injuries, common and superficial femoral artery injuries were most common (10/31). Arterial repair included interposition saphenous vein graft in seven patients, thrombectomy with end-to-end / lateral repair in twelve patients, vein patch in two patients, and arterial ligation in four patients. Six patients had arterial ligation as part of primary amputation. 3/21 (14.3%) patients had secondary amputation after attempted arterial vascular repair of an extremity. There were a total of 17 venous injuries, 13 managed by lateral suture repair and 4 by ligation. The median (range) hospital stay was 8 (1–76) days. 5 patients died (14%).

**Conclusions:**

Major vascular injuries occurred in 10% of hospitalized war-related injured patients. Our secondary amputation rate of extremities was 14%. The presence of a vascular surgeon within a military surgical team is highly recommended. Basic principles and techniques of vascular repair remain an essential part of training general surgeons because it may be needed in unexpected wars.

## Background

War is a type of collective violence which is defined as an instrumental use of violence by members of a group against another in order to achieve political, economic or social objectives [1]. The highest rates of war-related deaths are in the WHO African Region followed by parts of the WHO Eastern Mediterranean region. More than half a million people died during the first Gulf War (1980–1988) between Iraq and Iran [1].

Explosive weapons are designed to increase the number and energy of casing fragments leading to multiple penetrating wounds [2]. This is why vascular injuries are often associated with multiple trauma leading to high mortality unless prompt and appropriate surgical management is made. The evacuation time, climate, and availability of medical resources will impact the outcome of surgical management of war-injured patients [3]. Shortening the evacuation time in the prehospital setting reduced the war-related mortality [4-6], while prolonged evacuation resulted in high mortality [7].

Ideally, war injuries should be treated by surgeons having military surgery experience. In fact, civilian surgeons may find themselves trapped in wars practicing military surgery without prior training or experience in this field [4].

The mechanism and pattern of vascular injury will vary in the same community in war and peace. The commonest mechanism of injury in civilian practice in most parts of the world is road traffic collisions. We have found in a prospective cohort study that vascular injuries constituted 1.2% of all hospitalized motor vehicle collision trauma patients in a civilian setting [8]. However in countries with armed conflicts penetrating trauma causes most of the vascular injuries [9]. Interestingly, effects of war on vascular injuries extend after the war. Asfar et al. have shown that penetrating vascular injuries increased in civilian surgical practice after the Second Gulf War reflecting the aftermath of the Gulf War on Kuwait [10].

Mubarak Al-Kabeer Teaching Hospital is a 400 bed hospital located in the centre of Kuwait City. During the Second Gulf War, fighting occurred close to the hospital leading to a short evacuation time. This gave us a unique opportunity for treating vascular injuries in multiply severe injured patients similar to a front line field hospital. [4]. We aimed to study the biomechanism, pattern of injury, magnitude, and outcome of vascular injuries treated at Mubarak Al-Kabeer Teaching Hospital, Kuwait during the Second Gulf War and to highlight lessons learned from that period.

### Patients and methods

All war-related injured patients who had vascular injury and were treated at Mubarak Al-Kabeer Teaching Hospital from August 1990 to September 1991 were studied. During the study period Mubarak Al-Kabeer Teaching Hospital received more than 1100 war-injured patients out of whom 361 patients were admitted. Data were retrieved from the Gulf War Injury Database which was retrospectively collected. A special form was designed to collect the data. Data were coded and an Access Program was used to design the database.

Studied variables included age, gender, site of vascular injury, mechanism of injury, associated trauma, type of vascular repairs, and clinical outcome. Comminuted/complicated open fractures were primarily managed by external fixators. Data were analyzed with the PASW Statistics 18, SPSS Inc, USA. Data were presented as mean (SD), median (range) or numbers (%) as appropriate.

## Results

There were a total of 36 patients with major vascular injuries during the study period. This constituted 10% (36/361) of all war-related hospitalized patients while 32 (89%) were males. Their mean (SD) age was 29.8 (10.2) years. 21 (58%) were civilian and 15 (42%) were soldiers.

Majority of injuries were caused by bullets (47.2%) and blast injuries (47.2%) (Table 1). Thirteen patients were Iraqi (36%), 11 were Kuwaiti (31%) and 12 were from other nationalities. Eight patients (22%) presented with shock on arrival to the hospital.

**Table 1:** Mechanism of vascular injuries

**Cause of injury**	**Number**	**%**
Bullet injury	17	47.2
Blast injury	17	47.2
Stab wound	2	5.6
Total	36	100%

Table 2 shows the anatomical distribution of injuries. Majority of patients had head and neck injuries beside the extremity injuries. Only 2.8% had chest trauma.

**Table 2:** Distribution of injuries of patients having vascular war-related injuries, n = 36, August 1990 to September 1991, Mubarak Hospital, Kuwait

**Region**	**Number**	**%**
Head and neck	7	19.4%
Chest	1	2.8%
Abdomen and pelvis	3	8.3%
Upper limbs	8	22%
Lower limbs	21	58%

Type of arterial injury and their operative management are shown in Table 3. Injuries to the common femoral and superficial femoral arteries were most common (32%) followed by injuries to the popliteal arteries (19.4%) and brachial arteries (16.1%). Arterial repair included interposition saphenous vein graft in seven patients, thrombectomy with end-to-end / lateral repair in twelve patients, vein patch in two patients, and arterial ligation in four patients. Six patients had arterial ligation as part of a primary amputation. No prosthetic grafts were used in these patients. Types of venous injuries and their management are shown in Table 4. There were a total of 17 venous injuries. 13 were managed by lateral suture repair and 4 by ligation.

**Table 3:** Types and operative management of arterial injuries

**Artery**	**Vein graft**	**Vein patch**	**Primary repair**	**Ligation**	**Total**
Common femoral	3	1	2	1	7
Popliteal	1		3	2	6
Brachial		1	2	2	5
Superficial femoral	2	-	1		3
Tibial	-	-		2	2
Radial	-	-	1	1	2
Carotid	-	-	2	-	2
Subclavian	1	-	-	-	1
Ulnar	-	-	-	1	1
Epigastric	-	-	-	1	1
Iliac	-	-	1	-	1
Total	7	2	12	10	31

**Table 4:** Types and operative management of venous injuries

**Vein**	**Primary repair**	**Ligation**	**Total**
Popliteal	2	1	3
Internal jugular	1	1	2
Femoral	2	-	2
Subclavian	2	-	2
Superficial femoral	2	-	2
Inferior vena cava	2	-	2
Iliac	1	-	1
Pulmonary	1	-	1
Brachial	-	1	1
Tibial	-	1	1
Total	13	4	17

Amputation was performed in nine patients. Six patients underwent primary amputation for mangled extremities. These included, above knee amputation in two patients, below knee amputation in two patients and below elbow amputation in two patients.

All primary repairs, except two, were performed on the same day of injury. The exact time between vascular injury and surgery was unknown in majority of the cases. Three patients had secondary amputation after attempted vascular repair for 21 limbs (14.3%). One patient had a gunshot injury to the knee with multiple fractures, and popliteal artery, vein and nerve injuries. He underwent primary repair of the popliteal artery with end-to-end anastomosis and fasciotomy 24 hours after the injury. The patient subsequently developed thrombosis of the graft and limb ischemia which required above knee amputation. A 7-year-old boy was involved with a blast injury and transferred to our hospital from Iraq, underwent delayed primary repair of the femoral artery seven days after the injury. He had thrombectomy and end-to-end anastomosis but this ended with a below knee amputation because of delayed ischaemia. Another patient had a blast injury, underwent popliteal artery repair with interposition saphenous vein graft within six hours of injury. This was complicated by deep soft tissue infection and graft thrombosis that needed above knee amputation. The median (range) hospital stay of our patients was 8 (1–76) days. 5 patients died (14%).

## Discussion

Blast and bullet injuries caused majority of vascular injuries in our study. Most occurred in extremities and head and neck. The rarity of chest vascular injuries in our study is possibly related to early death of chest injury patients at the field before arrival to the hospital.

Common femoral, superficial femoral, and brachial arteries were the most common injured arteries in our study. This is similar to other reports. In Vietnam Vascular Registry, the superficial femoral and brachial arteries were the most common injured arteries [5]. Similarly, Fox et al. reported involvement of superficial femoral and brachial arteries in 44% of their cases [7]. Among 6808 reported vascular injuries in the literature, femoral artery injury was the most common (35%) followed by the brachial (31%) and then popliteal artery injuries (19.5%) [11]. Balad Vascular Registry from Iraq war included 90 femoral arteries and 44 popliteal arteries [12]. That is different from blunt vascular injuries caused by road traffic collisions in civilian practice, in which brachial artery is the most common injured vessel [8].

Arterial primary repair was the most common method of repair in our study (12/31). Only seven patients have their arterial repair performed with reversed saphenous vein graft. In contrast, most studies recommended using the interposition vein graft [7,13]. Experienced vascular and transplant surgeons were available through the whole war period in our hospital explaining the variation of techniques used in our study. Management of arterial repair with autologous vein graft remains the most durable and effective means of vascular repair [7,13]. Arterial injuries usually have a segmental arterial loss preventing tensionless primary anastomosis. Ligation of arterial injuries is a good strategy only in selected vessels. In our study, ligation of the radial, ulnar and tibial arteries did not cause ischaemia of the involved limbs. Examination of extremities after ligation is important to confirm limb viability. Prosthetic grafts were not used in any of our patients. Using prosthetic grafts remains a controversial issue because they are associated with increased risk of infection and consequently poor outcome [5,14].

Ligation of injured veins was commonly used during war [5,15]. However, in our series only four out of 17 venous injuries had ligation. This can be also explained by the presence of experienced vascular surgeons in our hospital. Venous repair remains a controversial issue in patients with vascular injuries. However, most would agree that venous repair by means, other than simple lateral suturing and end-to-end anastomosis, is a time- consuming process with uncertain benefits especially in multiply injured patients [5]. In our series most patients with venous injury underwent simple lateral repair or ligation if the first option was not possible.

Primary amputation was performed mainly because of mangled extremity with massive tissue loss, and bone injury, while secondary amputation was related to delayed presentation and infectious complications. Wani et al. treated 360 war-related arterial injuries over 13 years in Kashmir [16]. Their annual proximal peripheral arterial injuries were similar to ours. Nevertheless, their extremity amputation rate (less than 5%) was much less than ours (14%).

The decision for limb amputation is more difficult than it seems. We tried at the early period of the war to save as much limbs as we could but we learned later that this cannot be achieved all the time. Sometimes, early amputation can be the best option for some patients that saves their lives. Amputation rate depends on many factors including the severity of limb injury, mechanism of injury, ischaemia time, presence of associated injuries, and disaster situations when treating mass causalities [17].

It is a major principle in management of war-injured patients that saving a life comes before saving a limb. Mine injuries of the lower limbs are specifically more notorious and cause internal limb damage more than what appears on the skin. The blast injury of the mine causes high pressure that is transmitted proximally between the muscles causing major damage to the tissues.

We did not cover the vascular graft of the popliteal region with healthy viable tissue in two patients because of loss of all superficial tissues. We learned that this is a major problem that can lead to limb loss even with a successful graft because the graft has to be covered by viable tissue to prevent dehydration and infection. A rotational gastrocnemius flap if used to cover the popliteal vessels [18] could have possibly saved two secondarily amputated limbs having popliteal injuries in our series.

### Limitations of the study

The data of the present study is a historical data of our Gulf War Registry. Nevertheless, we think that it is very important to share this information with others. Civilian surgeons suddenly practicing war surgery without previous experience in this field tend to repeat the same old mistakes that surgeons learned from previous wars.

We could not define the exact time between vascular injury and surgery in majority of the cases. Nevertheless, we think that majority were operated within 6 hours of injury because fighting occurred very close to our hospital and the evacuation time was less than one hour [4]. There were no extensive diagnostic radiological procedures and wounds were explored in the operating theatre as soon as possible depending mainly on the clinical findings.

There have been many technical developments in the last two decade including principles of damage control surgery, use of portable ultrasound machines, and endovascular techniques. Despite that, we have recently noticed in the recent war conflicts in our region that most of these advanced techniques are not affordable except damage control surgery. Basic principles of using the least expensive surgical methods that help the maximum number of patients is still the major principle.

We did not use temporary vascular shunts for peripheral vascular injuries. Simple damage control surgery methods can reduce the operating time in mass casualty situations [19,20]. Rasmussen *et al.,* have used temporary vascular shunts in 30 extremities as a damage control adjunct in the Iraq war, especially for major proximal vascular injuries [21]. There were no shunt related complications, 86% were patent and only 7% needed early amputation [21]. This simple technique was useful to stabilize and then transport patients.

Ultrasound technology has dramatically evolved during the last two decades. New portable hand held ultrasound machines with excellent images and doppler color facility can be used in the battle field [22]. Duplex ultrasound has been successfully used to diagnose vascular injuries during the recent Iraq Conflict [17].

Angiography / Endovascular means was not used in our series. Therefore, it is possible that occult vascular injuries have been possibly missed and those usually present later [23]. The value of endovascular approach for both diagnosis and treatment of vascular injury in civilian and war practice is well studied [7,24,25] Fox et al. reported their experience of managing 107 soldiers with vascular injuries during the Iraq/Afghanistan wars [7]. They found that endovascular interventions resulted in lower morbidity and mortality in multiply injured patients.

## Conclusions

Major vascular injuries occurred in 10% of hospitalized war injured patients. The presence of vascular surgeons within a military surgical team is highly recommended. Basic principles and techniques of vascular repair remain an essential part of training general surgeons as it may be needed in unexpected wars.

## Competing interests

The authors declare that they have no competing interests.

## Authors’ contributions

AJ helped in the idea and design of the study, analyzed the data and wrote the manuscript. AA helped in the idea and analysis of the data and approved its final version. MN helped in the idea, drafting the first version of manuscript, and critically reading it. MA helped in the idea, and edited the manuscript. FAZ had the idea, designed the study protocol, collected and assessed the quality of the data, helped in writing the first draft of the paper, and repeatedly critically edited it. All authors have read and approved the final version of the manuscript.
